# High-Efficiency Wastewater Purification System Based on Coupled Photoelectric–Catalytic Action Provided by Triboelectric Nanogenerator

**DOI:** 10.1007/s40820-021-00695-3

**Published:** 2021-09-14

**Authors:** Shen Shen, Jiajia Fu, Jia Yi, Liyun Ma, Feifan Sheng, Chengyu Li, Tingting Wang, Chuan Ning, Hongbo Wang, Kai Dong, Zhong Lin Wang

**Affiliations:** 1 CAS Center for Excellence in Nanoscience, Beijing Key Laboratory of Micro-Nano Energy and Sensor, Beijing Institute of Nanoenergy and Nanosystems, Chinese Academy of Sciences, Beijing, 100083 People’s Republic of China; 2 Jiangsu Engineering Technology Research Centre for Functional Textiles, Jiangnan University, No.1800 Lihu Avenue, Wuxi, People’s Republic of China; 3 School of Nanoscience and Technology, University of Chinese Academy of Sciences, Beijing, 100049 People’s Republic of China; 4 School of Material Science and Engineering, Georgia Institute of Technology, Atlanta, GA 30332-0245 USA

**Keywords:** Triboelectric nanogenerators, Self-powered, 3DGA@CDs-TNs photocatalyst, Hybrid system, Wastewater purification

## Abstract

It is of great importance to explore a creative route to improve the degradation efficiency of organic pollutants in wastewater. Herein, we construct a unique hybrid system by combining self-powered triboelectric nanogenerator (TENG) with carbon dots-TiO_2_ sheets doped three-dimensional graphene oxide photocatalyst (3DGA@CDs-TNs), which can significantly enhance the degradation efficiency of brilliant green (BG) and direct blue 5B (DB) owing to the powerful interaction of TENG and 3DGA@CDs-TNs photocatalyst. The power output of TENG can be applied for wastewater purification directly, which exhibits a self-powered electrocatalytic technology. Furthermore, the results also verify that TENG can replace conventional electric catalyst to remove pollutants effectively from wastewater without any consumption. Subsequently, the unstable fragments and the plausible removal pathways of the two pollutants are proposed. Our work sheds light on the development of efficient and sustainable TENG/photocatalyst system, opening up new opportunities and possibilities for comprehensive utilization of random energy.

**Supplementary Information:**

The online version contains supplementary material available at 10.1007/s40820-021-00695-3.

## Highlights

- Triboelectric nanogenerator (TENG)/carbon dots-TNs supported over three-dimensional graphene aerogel (3DGA@CDs-TNs) system was constructed.
- TENG and 3DGA@CDs-TNs present excellent degradation activity toward brilliant green (88.26%, 40 min) and direct blue 5B (89.6%, 90 min).
- The coupling interaction of photocatalysis and TENG leads to an enhanced removal ability.
- The degradation pathways for brilliant green and direct blue 5B are proposed.

**Supplementary Information:**

The online version contains supplementary material available at 10.1007/s40820-021-00695-3.

## Graphical abstract

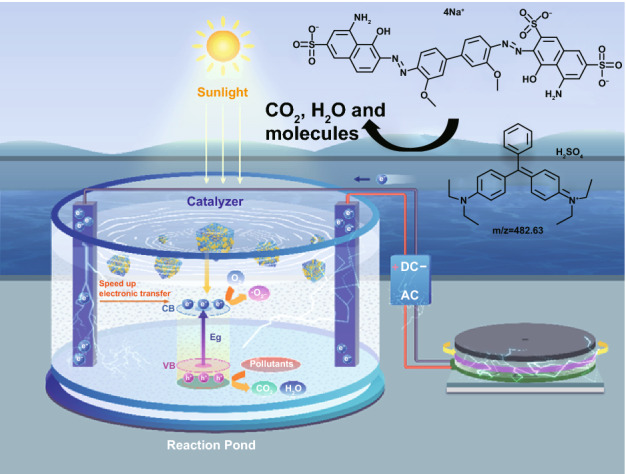

## Introduction

According to the World Wildlife Fund (WWF) and UNESCO (The United Nations Educational, Scientific and Cultural Organization), a large portion of water source has changed toward deterioration situation. Currently, under the water consumption trend, the two organizations have evaluated that the world population could suffer from water shortages by 2025 [1–3]. For wastewater, organic dyes created during the industries untreated emission are a major issue not only owning to the hazardous effect on aquatic quality but also due to their persistence, toxicity as well as carcinogenicity [4–6]. In particular, dyes can cause hindrance to the sunlight penetration and reduction in dissolved oxygen, which disturb ecological balance. For these reasons, removal and lowering of toxicity of organic dyes from industrial water are impending [7, 8].

Threatened by the scarcity of worldwide energy supply and ever-growing environmental crisis, an enormous challenge aiming to achieve better degradation efficiency of organic pollutants and high utilization of clean energy (blue energy, water energy, and wind energy) is extremely urgent [9–11]. Photocatalytic technique is a compelling strategy for aquatic and environmental remediation owning to its relatively low cost and robust chemical stability with no secondary pollution [12, 13]. Solar energy can stimulate catalysts to generate photoelectron holes and produce active radicals (^·^OH, ^·^O_2_^−^), which can destroy or oxidate organic matters [14–16]. Howbeit, due to the low solar energy utilization associated with easy recombination of e^−^-h^+^ pairs, the degradation efficiency of photocatalyst is limited [17–19]. Most important, the solar energy is not always available in environment, which makes photocatalystic degradation is not occur in the darkness. For this situation, construction of hybrids system is a key factor in improving overall efficiency [20–22]. Among various hybrids, photoelectric catalysis exhibits superior energy conversion efficiency and outstanding catalytic capacity [23–25]. In particular, owning to the interactions between the two routes, coupling of electrocatalysis with photocatalysis can enhance the amount of charge carriers and hinder the recombination of photogenerated electron–hole pairs [26–28]. However, traditional electrocatalysis confronts several defects, including large electricity consumption, harsh reaction conditions, and external power sources, which severely restrict its applications.

Triboelectric nanogenerator (TENG) with the ability of sustainable energy harvesting can provide an effective method for directly addressing the above issues [29–34]. As an alternative candidate, TENG may replace traditional electrocatalysis due to self-powered without any consumption and has no dependence on environmental conditions [35–38]. Although some related researches have been explored, there is no report about a system that consists of TENG and photocatalytic for purifying wastewater. The purpose of employing TENG is to use self-powered instead of electrocatalysis or other technologies to purify pollutants from wastewater [39–41]. TENG can capture and convert ambient motions into electrical energy based on the combination of triboelectrification and electrostatic induction, which has sparked extensive research for scavenging power [42–47]. Recently, TENG, as a promising self-powered manner toward harnessing blue energy, can be used for splitting water [48–50] and treating air pollutants [51]. Notably, energy from wind, solar and ocean waves has the potential to be integrated with electrical power grids to meet wastewater treatment requirements thanks to the infinite availability of renewable energy in nature [52].

Based on the above discussion, we construct a new working system to remove organic pollutants by combining TENG with photocatalysis technique. Carbon quantum dots-TiO_2_ sheets (CDs-TNs) catalysts were introduced into 3DGA structure (3DGA@CDs-TNs photocatalyst) by the addition of CDs-TNs during the hydrothermal synthesis of 3DGA. The interaction effect of 3DGA@CDs-TNs photocatalyst and TENG is analyzed by photoelectrocatalytic experiments. The pollutant degradation performance of TENG/3DGA@CDs-TNs system is superior to that of TENG and 3DGA@CDs-TNs photocatalyst alone, signifying TENG/3DGA@CDs-TNs has the potential used for wastewater remediation. In addition, possible pathways for the degradation of BG and DB are systematically proposed via the identification of fragments based on the identification of process fragments via liquid chromatography–tandem mass spectrometry (LC–MS/MS). Finally, the underlying degradation mechanism of the TENG/3DGA@CDs-TNs is also clarified.

## Materials and Methods

### Materials

Graphite powder (99.8%), tetrabutyl titanate (TBOT), Congo red, and citric acid were provided by Aladdin Reagent Company. Sodium nitrate (NaNO_3_), sulfuric acid (H_2_SO_4_), potassium permanganate (KMnO_4_), hydrochloric acid (HCl), hydrofluoric acid (HF), sodium hydroxide (NaOH), hydrogen peroxide (H_2_O_2_), brilliant Green (BR), and direct sky blue 5B (DB) were purchased from Sinopharm Chemical Reagent Co., Ltd, China (SCRC). Ammonium hydroxide was supplied by J&K Scientific Ltd. DI water from ULUPURE pure ultrapure water system was used in experiments.

### Preparation of CDs-TNs

TiO_2_ were prepared by hydrothermal method as depicted previously [53]. Typically, 3 mL of HF was added into 25 mL of TBOT with stirring. The mixture was placed into a Teflon-lined autoclave and kept at 180 °C for 24 h. The products were collected and filtered by NaOH and DI water, and then by an oven. 1.051 g of citric acid was dissolve into 25 mL of DI water, and the pH value of solution was adjusted to 6 by using NH_4_OH. After that, 0.4 g of TNs were added into the above mixture. The solution was sealed in a Teflon-lined autoclave at 160 °C for 4 h. Afterward, the precipitates were collected by centrifugation and washed with DI water and then dried at 60 °C for 12 h.

### Preparation of 3DGA@CDs-TNs Photocatalyst

Graphene oxide (GO) was synthesized through the modified Hummers’ method [54]. In a briefly approach for the synthesis of 3DGA@CDs-TNs photocatalyst, 20 mg of as-prepared CDs-TNs was added into 20 mL of GO dispersion (6 mg mL^−1^) to obtain a uniform mixture. The mixture was sonicated and stirred for 1 h. Then the mixture was transferred into 100 mL Teflon-lined autoclave and maintained at 180 °C for 12 h to synthetize 3DGA@CDs-TNs photocatalyst. Finally, the prepared 3DGA@CDs-TNs photocatalyst was purified with DI water and freeze-dried for 12 h. The 3DGA aerogel was synthesized via the same approach as described above without adding CDs-TNs catalysts.

### Fabrication of TENG

The framework of the TENG is separated into two components: a rotator and a stator. For the stator: (1) An acrylic plate with the thickness of 3 mm and the diameter of 20 cm was employed to form a square-shaped substrate using a laser cutter. (2) A layer of soft foam was pasted on the acrylic acts as buffer layer. (3) A layer of conductive fabric was adhered onto the soft foam. The conductive fabric as electrode was patterned using a laser cutter (diameter: 14.4 cm) and had complementary as well as separated sectors. (4) A layer of PTFE as one of the triboelectric layer was pasted on the conductive fabric. For the rotator: (1) A disk-shaped acrylic substrate (diameter: 14.4 cm, thickness: 3 mm) was shaped through a laser cutter, which consisted of radially arrayed sectors with a central angle of 18°. (2) A copper layer was stuck on the side of acrylic substrate as friction layer, which possesses the same shape as that of one electrode.

### Characterization

Thechemical states of as-prepared samples were characterized by an X-ray diffraction (XRD, Bruker AXS) under Cu Kα radiation with diffraction angle of 10° to 90°. The microstructures and morphological structures were captured through scanning electron microscopy (SEM) by su1510 microscope and transmission electron microscopy (TEM) by JEM-2010 electron microscope (JEOL). The electrochemical impedance spectroscopy (EIS) and photocurrent response measurements were performed by using Zennium PP211 electrochemical workstation. The output voltage and current of the TENG were monitored by an electrometer (Keithley 6514).

The LC–MS technique was employed to identify the decomposed intermediates and final products of BG and TC (Waters, USA); BEH C18 column (1.7 μm, 2.1 × 150 mm). The detailed works are given in supporting information.

### Organic Pollutants Degradation

Photocatalytic removal experiment: the visible light irradiation was achieved by a 500 W (XO-CHX-ID) Xe lamp with a 420 nm cutoff filter. Briefly, 10 mg of as-prepared catalysts was suspended in 30 mL of BG (5 ppm) and DB (20 ppm) aqueous solution, and the solution was stirred in the darkness to ensure the adsorption–desorption equilibrium. At preset intervals, the reaction solution was collected and filtered to remove catalysts. The absorbance of BG and DB in supernatant was examined by UV–Vis spectrophotometer.

Self-powered integration system for pollutants removal: all the experiments for degrading BG and DB were carried out in a breaker containing 30 mL solution of BG and DB. 10% of NaCl solution was selected as electrolyte. Graphite and iron acting as cathode and anode were immersed into the reaction container. A rectifying bridge was used to convert the alternating current to direct current.

## Results and Discussion

### Structure and Output Performance of TENG

**Fig. 1:**
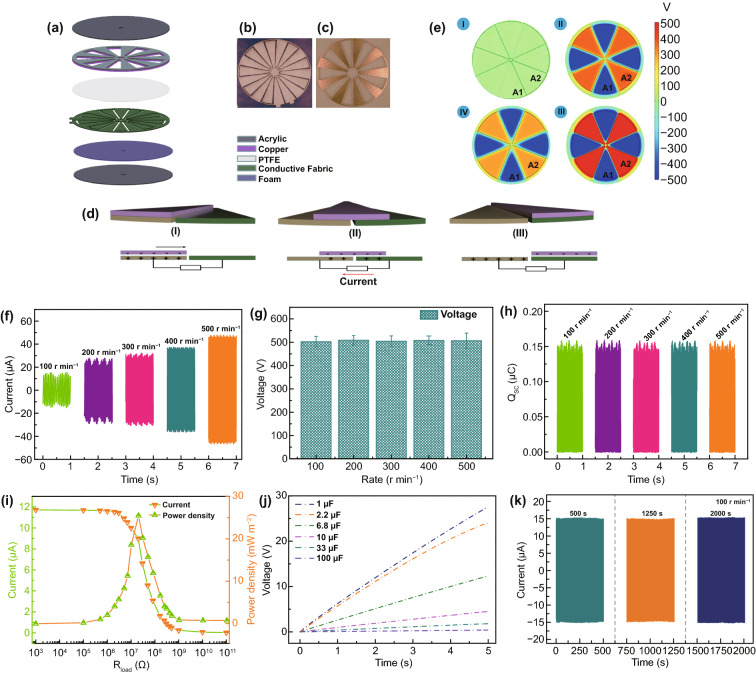
Schematic diagram. **a** A schematic illustration of the functional components of multifunctional TENG. A photograph of **b** stator and **c** rotator. The working principles of TENG with the freestanding module: **d** schematic working principle of TENG (I–III) and **e** the simulated potential distributions for TENG at three different rolling displacements by COMSOL software. Performance of TENG. **f** Short-circuit current, **g** open-circuit Voltage, **h** short-circuit charge quantity of TENG at different rotation rates. **i** Capacitor charging ability of the TENG under 100 r min^−1^. **j** Current and peak power of the TENG measured with different external load resistances under 100 r min^−1^. **k** Stability of open-circuit voltage over 16,000 cycles

The multifunctional structure of TENG is displayed in Fig. 1a, which is mainly composed of two parts: a rotator and a stator. The rotator consists of an acrylic sheet with uniformly distributed 9 of Cu layers. Each of radial array sectors has a length of 7.2 cm and central angle of 18°. And the Cu layer acts as contact–triboelectrification surface. For the stator, it can be divided into four parts: a layer of polytetrafluoroethylene film (PTFE) as an electrification material, a layer of conductive fabric deposited on the soft foam as the electrodes and an acrylic sheet as the underlying substrate. In particular, the electrode layer is constituted by two adjacent complementary electrode frameworks which are separated by gaps. In addition, the photographs of the rotator and the stator are presented in Fig. 1b, c. The working mechanism of TENG is illustrated in Fig. 1d. In the original state, owning to the different triboelectric polarities of the two contact materials, positive and negative charges can be produced on the Cu surface and PTFE film, respectively, after triboelectrification (step i). Once the rotator sweeps across the stator, charges are injected from Cu layer to the PTFE film due to the conservation of charge (step ii). Once the Cu electrode overlaps the electrode B, all the electrons are flowed to the electrode B (step iii). As visualized in Fig. 1e, numerical calculation results further demonstrate the electrostatic potential difference of TENG in the contacting state and separating state.

The electrical output performance of TENG including open-circuit voltage (*V*_OC_), short-circuit current (*I*_SC_), and short-circuit charge transfer (*Q*_SC_) with variable rotating speeds is presented in Fig. 1f–h. The *I*_SC_ of TENG is gradually enhanced with the increase in the rotating speed. As the rotating speed increases from 100 to 500 r min^−1^, the *I*_SC_ increases from 15.6 to 48.2 μA (Fig. 1f), suggesting that the higher rotating speed can enhance the output current. The *V*_OC_ with different rotating speeds are shown in Fig. 1g. As depicted, the *V*_OC_ is saturated at about 510 V. Moreover, with increasing the rotating speed, no obvious change of the *V*_OC_ is found, which indicates that the increase in the rotating speed has no effect on the *V*_OC_. In addition, there is no enhance for *Q*_SC_ with the speed accelerates, and a steady value at about 0.153 μC is observed (Fig. 1h).

To evaluate the output power of TENG, the output current was tested by connecting it with different external resistances (Fig. 1i). At a rotator rate of 100 r min^−1^, the output current drops with the external resistances increase. The output power achieves the maximum peak of 25.48 mW m^−2^ rapidly at a resistance of 20 MΩ. Furthermore, a series of capacitors (1–100 μF) are employed to demonstrate the charging capability of TENG. As shown in Fig. 1j, the capacitor (1 μF) is charged to 27.8 V in 5 s under the rotator rate of 100 r min^−1^. With increasing the capacitance, the voltage of capacitors clearly decreases and a sluggish charging process is occurred.

Robust stability is a critical concern for TENG in practical application [55]. Thus, the output performance of TENG was measured under 100 r min^−1^ during 2000s consecutive rotation. As shown in Fig. 1k, the voltage of TENG exhibits a stable trend and an unobservable variation, proving a favorable stability of TENG.

### Structure and Electrochemical Performance of 3DGA@CDs-TNs Photocatalyst

**Fig. 2:**
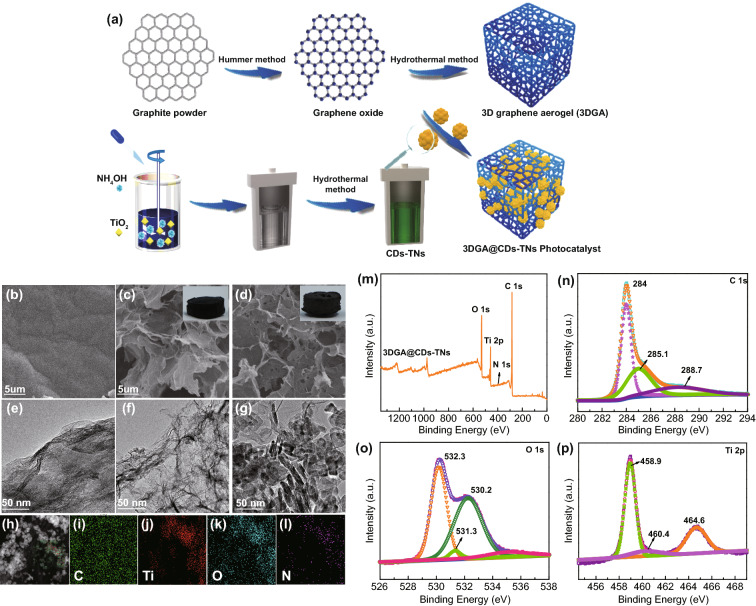
**a** Schematic showing the synthesis of 3DGA@CDs-TNs photocatalyst. SEM images of **b** GO, **c** 3DGA inset: the picture of 3DGA; and **d** 3DGA@CDs-TNs, inset: the picture of 3DGA@CDs-TNs. TEM image of **e** GO, **f** 3DGA, and 3DGA@CDs-TNs **g**, and corresponding EDS. **h–l** mapping images for survey, C, N, O, and Ti in 3DGA@CDs-TNs photocatalyst. XPS spectra of 3DGA@CDs-TNs photocatalyst. **m** Survey spectrum, **n** C 1s, **o** O 1s, and **p** Ti 2p

Figure 2a shows the synthesis of 3DGA@CDs-TNs photocatalyst. The microstructures of samples were captured by SEM and TEM. It is shown that GO has smooth surface with a 2D structure. As displayed in the insets of Fig. 2b, c, the 3DGA@CDs-TNs photocatalyst appears smaller than the blank 3DGA. Simultaneously, the structure of 3DGA and 3DGA@CDs-TNs photocatalyst shows the large appearance area and presents spatial network with cross-linked pores composing by randomly dispersed GO nanosheets. Figure 2e displays an ultrathin sheets and irregular creased of GO. The TEM image of 3DGA shows a folding structure (Fig. 2f). Figure 2g exhibits the TEM image of 3DGA@CDs-TNs photocatalyst, from which it can be observed that CDs-TNs catalysts are well dispersed on the surface of 3DGA. In addition, the synthesized CDs are spherical and well dispersed (Fig. S1a). Meanwhile, the TEM image of 0.2CDs/TNs (Fig. S1b) exhibits CDs uniformly distributed on the TNs surface. EDS mapping images (Fig. 2h-l) of 3DGA@CDs-TNs photocatalyst further demonstrate the existence of C, N, O, Ti elements in 3DGA@CDs-TNs photocatalyst.

The survey XPS spectrum of 3DGA@CDs-TNs photocatalyst further reveals the presence of C, N, O, Ti elements. In addition, the respective atomic content is 69.65%, 1.18%, 24.38%, and 4.79% (Fig. 2m and Table S1). For the C 1s region (Fig. 2n), the signal can be divided into three peaks at 284, 285.1, and 288.7 eV, corresponding to C=C/C–C, C-Ti–O, and C=O. According to the Fig. 2o, the O 1s band is fitted to four peaks at 530.2, 531.3, and 532.3 eV, which are related to the occurrence of C=O, C–OH, and O-Ti–O. Figure 2p shows the Ti 2p carves of 3DGA@CDs-TNs photocatalyst, which can be deconvoluted into three peaks (458.9, 460.4, and 464.6 eV) including two peaks of Ti 2p3/2 and the peak of Ti 2p1/2. XPS results confirm the electronic interaction between CDs-TNs and 3DGA in 3DGA@CDs-TNs photocatalyst.

**Fig. 3:**
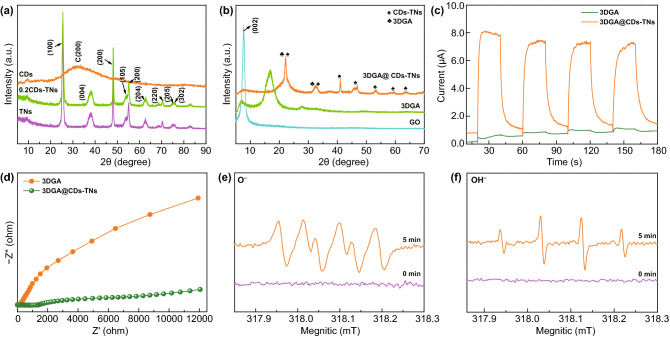
XRD patterns of samples: **a** CDs, TNs, and CDs-TNs, **b** GO, 3DGA, and 3DGA@CDs-TNs photocatalyst. **c** EIS curves and **d** transient photocurrent densities for 3DGA@CDs-TNs photocatalyst under intermittent visible light irradiation. ESR spin-trapping profiles for **e** DMOP-⋅O_2_^−^ and **f** ⋅OH generation over 3DGA@CDs-TNs photocatalyst

Figure 3a, b illustrates the XRD patterns of samples. The distinct diffraction peak at 30.5° for CDs is index to (200) disordered graphite-like species. For TNs and CDs/TNs composites, all characteristic peaks from 25.28° to 75.03° correspond to the crystal planes of anatase TiO_2_ (PDF No. 00–021-1272). Nevertheless, for CDs-TNs composites, no characteristic diffraction for CDs is observed, owing to the limited content of CDs in the latter. GO sample shows the distinct peak at 7.7°, owing to the (002) plane of GO. After hydrothermal route, the peak of GO almost disappears and a broad distinct peak of 3DGA occurs at 16.6°, indicating that GO is converted into rGO successfully by hydrothermal process. For 3DGA@CDs-TNs photocatalyst, the peaks are dramatically shifting to high angle due to the existence of CDs-TNs catalysts on 3DGA structure, which expand the interlayer spacing. Moreover, Fig. S2 shows the XRD patterns of samples after reaction. It can be seen that there are no noticeable changes in the crystal structure for 3DGA@CDs-TNs before and after degrading, which is further verifies the robust stability of 3DGA@CDs-TNs.

Transient photocurrent response and EIS measurements reveal the charge transfer ability of 3DGA@CDs-TNs photocatalyst [56]. From Fig. 3c, the current intensity for 3DGA@CDs-TNs photocatalyst is significantly increased after introducing CDs-TNs. Obviously, a narrower arc radius (Fig. 3d) is observed for 3DGA@CDs-TNs photocatalyst under visible light simulation with respect to that of 3DGA, which demonstrates that the loading of CDs-TNs can facilitate charge transport and separation, resulting in a superior degradation capacity of 3DGA@CDs-TNs photocatalyst. Moreover, CDs/TNs show a dramatically enhanced photocurrent response and a smaller semicircle diameter (Fig. S3a, b), revealing that CDs can accelerate the migration rate of e^−^-h^+^ pairs, thereby contributing to the remarkable photodegradation performance. Additionally, electron spin resonance (ESR) technique was also carried out for detecting DMPO-O_2_^−^ and DMPO-OH. As illustrated in Fig. 3e, f, no signal of DMPO-·OH is monitored under the darkness in the existence of 3DGA@CDs-TNs photocatalyst. After irradiating for 5 min, four typical peaks of ·OH are observed. Similarly, no characteristic peaks of ·O_2_^−^ are observed in the absence of visible light. Since irradiated to visible light, four obvious signals of ·O_2_^−^ can be captured, which verify that the H_2_O and O_2_ in the reaction system can be converted into ·OH and ·O_2_^−^ by catalyst under visible light irradiation, thus degrading hazardous pollutants directly.

### Degradation Performance of TENG, 3D3DGA@CDs-TNs and TENG/3DGA@CDs-TNs

**Fig. 4:**
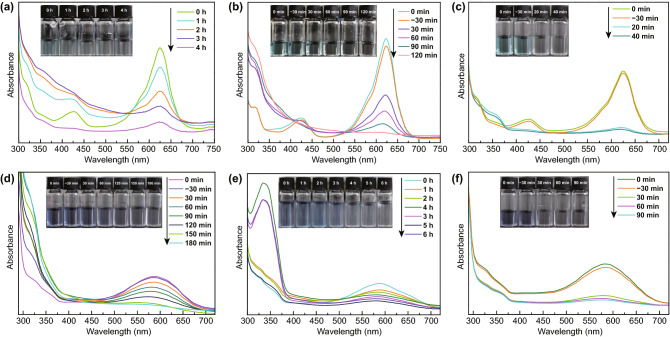
Changes of the characteristic absorption after degradation by 3DGA@CDs-TNs photocatalyst, TENG, and TENG/3DGA@CDs-TNs of **a–c** BG and photographs of residual BG solution (inset); **d–f** DB and photographs of residual DB solution (inset)

The degradation performance of TENG and photocatalyst are assessed by decomposing two representative structure pollutants: azo dye-DB and triphenylmethane dye-BG (Fig. 4). The characteristic absorbance of BG solution changed via different approach (TENG, 3DGA@CDs-TNs photocatalyst and TENG/3DGA@CDs-TNs) at different reaction times is shown in Fig. 4a–c. The results display the absorption intensities of BG decrease more rapidly with increasing the reaction time. The absorption peak at 624 nm seems to be completely disappeared with degradation time extending, verifying that BG is almost removed by TENG, 3DGA@CDs-TNs photocatalyst, and TENG/3DGA@CDs-TNs, respectively. Meanwhile, the peak at about 425 nm increases firstly and then decreases, revealing that organic fragments are decomposed to small molecules. Significantly, in the case of TENG/3DGA@CDs-TNs experiment, the absorption peak dips quickly in the first 20 min of degradation and is flat after 40 min, highlighting that BG degradation efficiency highly depends on the presence of TENG and 3DGA@CDs-TNs photocatalyst. The inset photographs in Fig. 4a–c indicate that BG aqueous solution is almost colorless by degradation. The spectrum resulting from the three methods of DB are portrayed in Fig. 4d–f. With reaction time prolong, the main peak at about 586 nm decreases. The color of DB solution almost disappears after decomposition, indicating the completely destruction of DB conjugated structure.

**Fig. 5:**
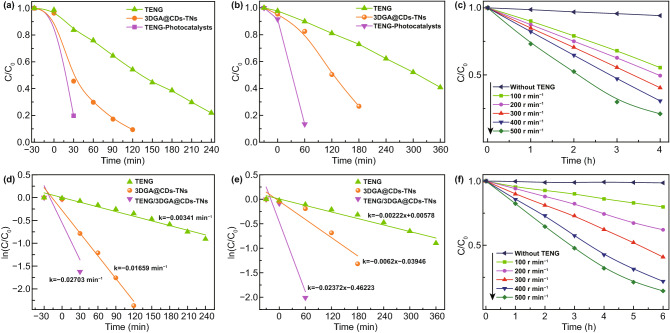
Degradation and degradation kinetics curves of BG (**a**, **b**) and DB (**c**, **d**) for 3DGA@CDs-TNs photocatalyst, TENG, and TENG/3DGA@CDs-TNs. Degradation of **e** BG, **f** DB by TENG with different rotation rates

To directly compare the degradation rates of pollutants over the three methods, the removal efficiency and the kinetics of degradation are also concluded (Fig. 5a–d). For the TENG experiment, the results show that 59.59% of BG can be degraded effectively under the rotator speed of 300 r min^−1^. Moreover, 3DGA@CDs-TNs photocatalyst exhibits the removal efficiency of 81.66% (BG) after 2 h visible light irradiation. Dramatically, in the existence of TENG and 3DGA@CDs-TNs photocatalyst, remarkable enhancements (88.26%, 40 min) in the decomposition of BG are observed. TENG, 3DGA@CDs-TNs photocatalyst, and TENG/3DGA@CDs-TNs display favorable effect on the decomposition of DB, in which degradation rates are 59.2% (6 h), 73.23% (3 h), and 89.6% (1.5 h), respectively (Table S2). As shown in Fig. 5a, b, the combination of TENG and 3DGA@CDs-TNs photocatalyst results in a higher capacity of pollutants degradation. This phenomenon is ascribed to that TENG provides bias potentials and current which can build an electric field and accelerate the separation and transfer of carriers, leading to an increased degradation efficiency. Furthermore, 3DGA exhibits the merits of electron collection and transportation and can efficiently hinder the recombination of photogenerated charges in hybrid process. The combination of TENG and 3DGA@CDs-TNs photocatalyst endows the TENG/3DGA@CDs-TNs system an interesting candidate for pollutants degradation in sunlight and ocean condition.

In addition, the kinetics of degradation under different conditions are expressed in the following equation:

1 $$\ln \left( {C_{t} /C_{0} } \right) = - kt$$

where *C*_0_, *C*_*t*_, and *k* are the initial equilibrium concentration, actual concentration of dyes at reaction time t, and rate constants, respectively. The relationship between ln(*C*_*t*_/*C*_0_) and t over TENG, 3DGA@CDs-TNs photocatalyst, and TENG/3DGA@CDs-TNs is plotted in Fig. 5c, d. From the result, it can be seen that the degradation of DB and BG follows the pseudo-first-order kinetics. The degradation kinetic parameters of TENG, 3DGA@CDs-TNs photocatalyst and TENG/3DGA@CDs-TNs are summarized in Tables S3 and S4. It can be seen that the TENG/3DGA@CDs-TNs system has the highest reaction rate constants of 0.01011 and 0.01338 for BG and DB dyes, respectively. The results illustrate the TENG-3DGA@CDs-TNs system achieves the highest degradation of pollutants compared to the TENG and 3DCA@CDs-TNs photocatalyst due to superior cooperation of photoelectron.

Figure 5e, f demonstrates the removal efficiency of the two pollutants at different rotation rates. As shown in Fig. 5e, f, 500 r min^−1^ has the fastest degradation rate, while 100 r min^−1^ has the slowest. Concretely, as the speed rate increases from 100 to 500 r min^−1^, the degradation rates for DB and BG increase from 20.07%, 40.61% to 77.8% and 85.55%, respectively (Fig. S4a, b). The increased degradation rates illustrate that the higher rotation rate enhances the degradation capability of TENG.

In Table 1, the degradation performance of TENG/3DGA@CDs-TNs is compared with other techniques reported previously. Obviously, taking into account the different experimental parameters, TENG/3DGA@CDs-TNs exhibits outstanding degradation activity among these methods.

**Table 1:** Comparison of pollutants degradation over different techniques

Technique	Light source	[Pollutants] (mg L^−1^)	[Catalyst] (g L^−1^)	Removal (%)	Refs.
Photo	Xe300W	Congred, 50	0.2	~ 90 (1 h)	[7]
Photo	40 W UV	Congred, 30	0.5	78 (2 h)	[9]
Photo	Visible300	RhodamineB, 10	0.5	~ 97 (2 h)	[10]
Photo	two EDMLs	RhodamineB, 10	1	~ 90 (1 h)	[11]
Fenton	–	4-nitrophenol, 10	–	91 (1 h)	[20]
Fenton	–	Orange II, 30	–	90 (20 min)	[21]
Fenton	–	Phenol, 30	–	80 (50 min)	[22]
Photo-Fenton	–	Phenol, 30	1	90 (50 min)	[22]
Photo-Fenton	Xe350W	Methylene B, 30	1 L	88 (50 min)	[23]
Photo-Fenton	Xe350W	Ibuprofen, 20	0.5	90 (60 min)	[24]
Photo-Fenton	Xe300W	Tetracycline, 20 DB,	0.4	62.7 (40 min)	[25]
TENG-photo	Xe300W	20 BG, 5	0.5	89.5 (1.5 h)	This work
	Visible500W			88.26 (40 min)	This work

### Degradation Pathway and Mechanism

**Fig. 6:**
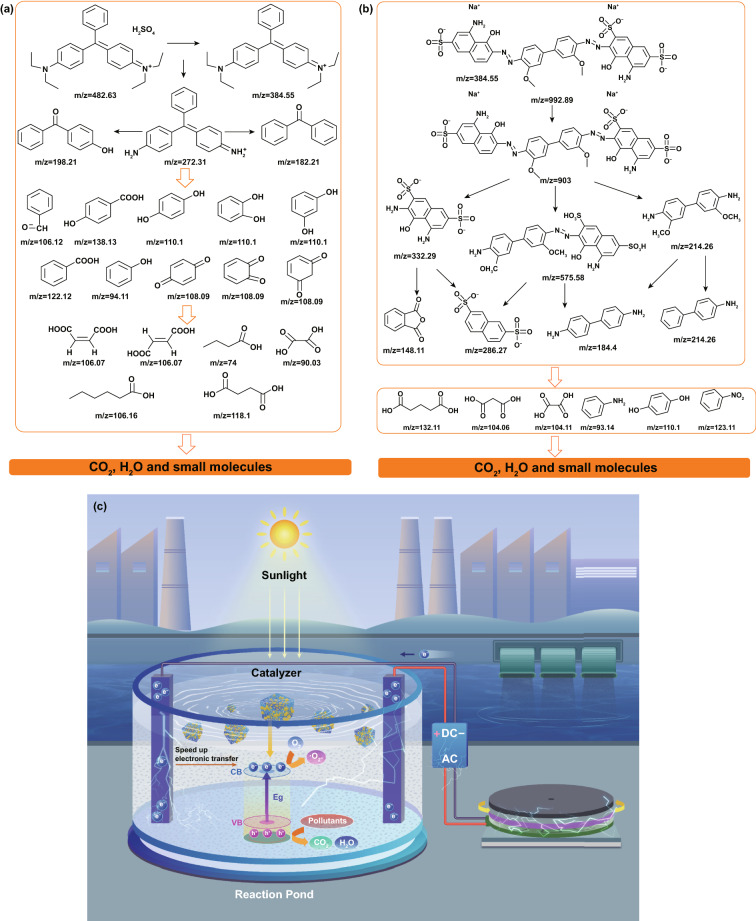
Possible pathways for the degradation of **a** BG and **b** DB over TENG/3DGA@CDs-TNs photocatalysts under visible light irradiation. **c** Schematic showing the degradation mechanism of TENG/3DGA@CDs-TNs

Notwithstanding the pollutants can be removed by TENG and 3DGA@CDs-TNs photocatalyst, hazardous fragments produced during the reaction process may also bring about environmental compounds [57]. Hence, LC–MS technique was employed to identify the transformation intermediates and final products of DB and BG. Possible small molecules and relative details regarding DB and BG are listed in Tables S5 and S6. According to the LC–MS results, fifteen intermediates of DB are deduced. As shown in Fig. 6a, product with *m*/*z* = 905 is derived from the initial DB, fragments with *m*/*z* = 332.29 and *m*/*z* = 214.26 are further produced by the breaking of –N=N–, and *m*/*z* = 575.58 is assign to the cleavage of –C–C– bond. And *m*/*z* = 575.58 can form *m*/*z* = 286.27 and *m*/*z* = 184.24 (benzidinel) via the breaking of –N=N–. Intermediates with *m*/*z* = 148.11 (o-phthalic anhydride) and *m*/*z* = 214.26 (4-aminobiphenyl hydrochloride) are owning to the oxidation of *m*/*z* = 332.29 and 214.26, respectively. Contaminants with *m*/*z* = 93.14, *m*/*z* = 110.1, and *m*/*z* = 123.11 can be attributed to the oxidation of *m*/*z* = 184.24 and ring opening of *m*/*z* = 148.11. Subsequently, products with *m*/*z* = 104.06, *m*/*z* = 104.11, and *m*/*z* = 132.11 are resulted from the mineralization of benzene ring. Based on the intermediates and final products analysis, the feasible decomposition pathway for DB is deduced.

Furthermore, the plausible removal pathway for BG is also proposed (Fig. 6b). Briefly, the ethyl group on the maternal BG is firstly attracted by free radicals, transforming the compound of *m*/*z* = 272.31. Moreover, fragments *m*/*z* = 198.21, *m*/*z* = 182.21, and *m*/*z* = 106.12 are assigned to the cleavage of *m*/*z* = 272.31. Then, the opening ring and mineralization reaction is emerged, and small molecules are produced by the breakdown of BG and the N-de-ethylated fragments.

In summary, the initial subtracts and unstable intermediates may be decomposed to CO_2_, H_2_O, and other small molecules.

Based on the principle of catalysis and our experiments, the photoinduced charge generation, separation, and transport in pollutants degradation as well as the mechanism of photoelectric catalysis are proposed (Fig. 6c). On the one hand, under visible light excitation, CDs can absorb visible light and emit UV light, which excites TNs to produce electron–hole pairs. The electrons can transit toward 3DGA and shuttle efficiently from CDs-TNs catalyst to aerogel, thus prolonging charge carrier lifetime and inhibiting the recombination of electron–hole pairs. On the other hand, when TENG is applied to the degradation process, the current of TENG can enhance the concentration of carriers because of the injection of electrons. Meanwhile, the applied bias can accelerate the separation of electron–hole pairs and hinder the rapid decay of photocurrent. In other words, the visible light simulation is used to induce the excitation within the semiconductor, while the applied bias, as an external driving force, is employed to promote the continuous separation of electron–hole pairs. Then, the electrons consume the superficial oxygen to generate superoxide (·O_2_^−^), which is taken part in the degradation process. Moreover, the holes of photocatalyst combine with H_2_O or OH^−^ to form ·OH, which directly decompose the organic pollutants.

## Conclusion

This study develops a novel TENG coupled with 3DGA@CDs-TNs system. The designed TENG can yield a high voltage of ~ 510 V and a high power density of 25.48 W m^−2^. The realization of synergetic photoelectric catalysis further increases the efficiency of photocatalyst in dyes degradation. The hybrid degradation system presents 6.65-fold and 5.74-fold enhancements for photoelectrocatalytic degradation of BG and DB compared to that of TENG. Besides, the kinetics of degradation of BG and DB over TENG/3DGA@CDs also exhibit 2.81-time and 1.99-time higher than those of 3DGA@CDs-TNs photocatalyst. The enhancement can be ascribed to the strong interaction TENG and 3DGA@CDs-TNs photocatalyst, which is due to that the TENG can provide photogenerated electrons and applied bias, promoting the separation and transfer of electrons and holes. Based on the results of LC–MS, the feasible degradation pathways for BG and DB are also clarified. This compelling strategy of converting mechanical energy and solar into sustainable energy sparks an inspiration in designing environmental-friendly photoelectrocatalytic system with excellent wastewater remediation performance.

## Supplementary Information

Below is the link to the electronic supplementary material.

Supplementary file1 (PDF 631 kb)
