# Supplementary material for: High-Efficiency Wastewater Purification System Based on Coupled Photoelectric–Catalytic Action Provided by Triboelectric Nanogenerator
Source: Nanomicro Lett. 2021 Sep 14;13:194. doi: 10.1007/s40820-021-00695-3 (PMC8440689; doi:10.1007/s40820-021-00695-3)
Supplement: Supplementary file 1 — Supplementary file1 (PDF 631 kb) [file 40820_2021_695_MOESM1_ESM.pdf]

Supporting Information for

High Efficiency Wastewater Purification System Based on Coupled Photo-Electric-Catalytic Action Provided by Triboelectric Nanogenerator

Shen Shen [1, 2, #], Jiajia Fu [2, #], Jia Yi [1], Liyun Ma [1], Feifan Sheng [1], Chengyu Li [1], Tingting Wang [1], Chuan Ning [1, 3], Hongbo Wang [2, *], Kai Dong [1, 3, *], Zhong Lin Wang [1, 3, 4, *]

[1] CAS Center for Excellence in Nanoscience, Beijing Key Laboratory of Micro-Nano Energy and Sensor, Beijing Institute of Nanoenergy and Nanosystems, Chinese Academy of Sciences, Beijing 100083, P. R. China

[2] Jiangsu Engineering Technology Research Centre for Functional Textiles, Jiangnan University, No.1800 Lihu Avenue, Wuxi, P.R. China

[3] School of Nanoscience and Technology, University of Chinese Academy of Sciences, Beijing, 100049, P. R. China

[4] School of Material Science and Engineering, Georgia Institute of Technology, Atlanta, GA 30332-0245, USA

[#] Shen Shen and Jiajia Fu contributed equally to this work.

*Corresponding authors. E-mail: wxwanghb@163.com (Hongbo Wang); dongkai@binn.cas.cn (Kai Dong); zhong.wang@mse.gatech.edu (Zhong Lin Wang)

Supplementary Tables and Figures

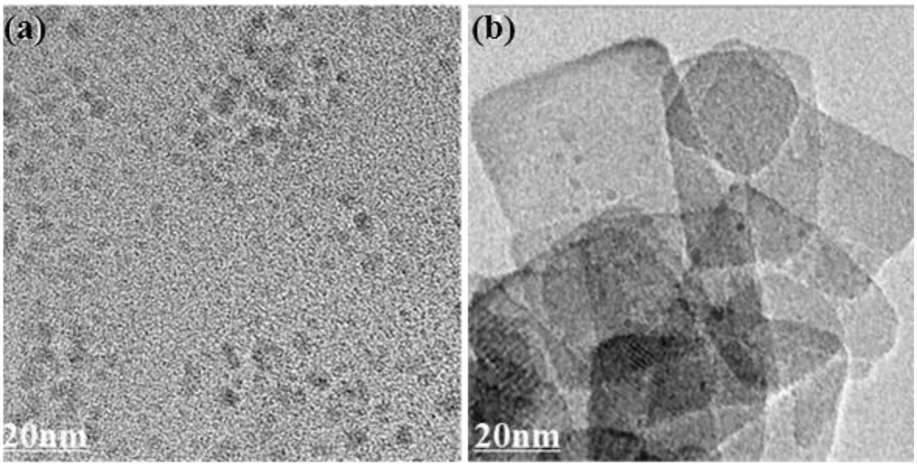

Fig. S1 TEM images of CDs (**a**) and 0.2CDs/TNs (**b**)

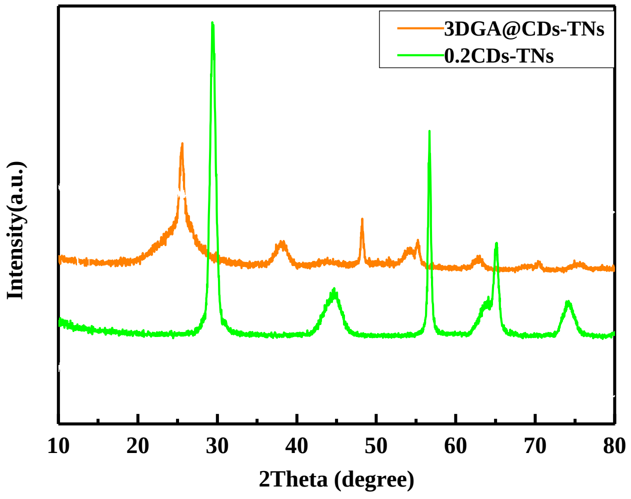

Fig. S2 XRD patterns of 3DGA@CDs-TNs and CDs-TNs after reaction

Table S1 Respective atomic content of 3DGA@CDs-TNs

Elements	Atomic %
C	69.65
N	1.18
O	24.38
Ti	4.79

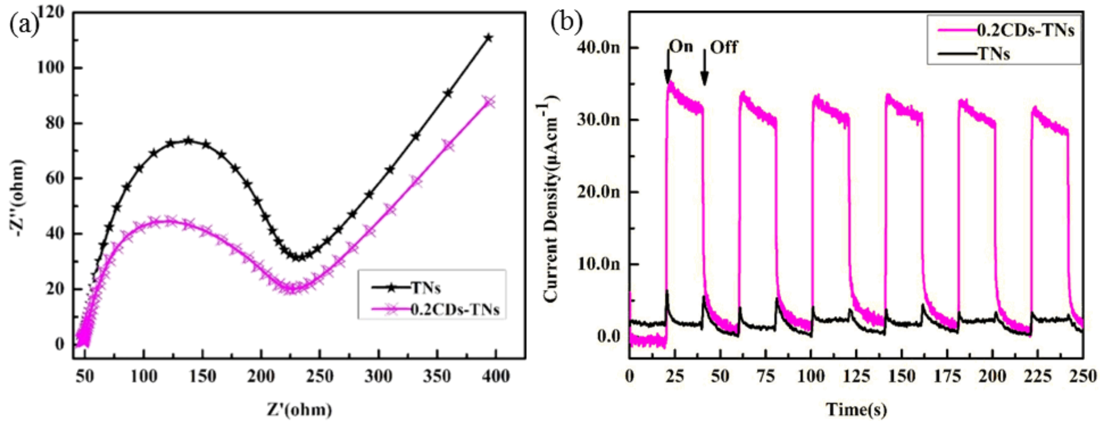

Fig. S3 EIS curves (**a**) and transient photocurrent density (**b**) of TNs and 0.2CDs/TNs under visible light irradiation

Table S2 Comparison of BG and DB degradation over different methods

Dyes	Ppm	E_{TENG}	$E_{Photocatalysts}$	$E_{TENG-3DGA@CDs-TNss}$
DB	5	59.2% (6h)	73.23% (3h)	89.6% (1.5h)
BG	20	59.59% (4h)	81.66% (2h)	88.26% (40min)

Table S3 Degradation kinetic parameters of BG over 3DGA@CDs-TNs, TENG and TENG/3DGA@CDs-TNs

Methods	k(10^{-3}/min)	Formula	R^2
TENG	3.41	y=-0.00341x+0.0009847	0.96148
3DGA@CDs-TNs	16.59	y=-0.01659x-0.27983	0.96638
TENG-Photocatalysts	27.03	y=-0.02703x-0.55186	0.53102

Table S4 Degradation kinetic parameters of DB over 3DGA@CDs-TNs, TENG and TENG/3DGA@CDs-TNs

Methods	k(10^{-3}/min)	Formula	R^2
TENG	2.22	y=-0.00222x+0.00578	0.95317
3DGA@CDs-TNs	6.2	y=-0.0062x-0.03946	0.88643
TENG-Photocatalysts	23.72	y=-0.02372x-0.46223	0.83139

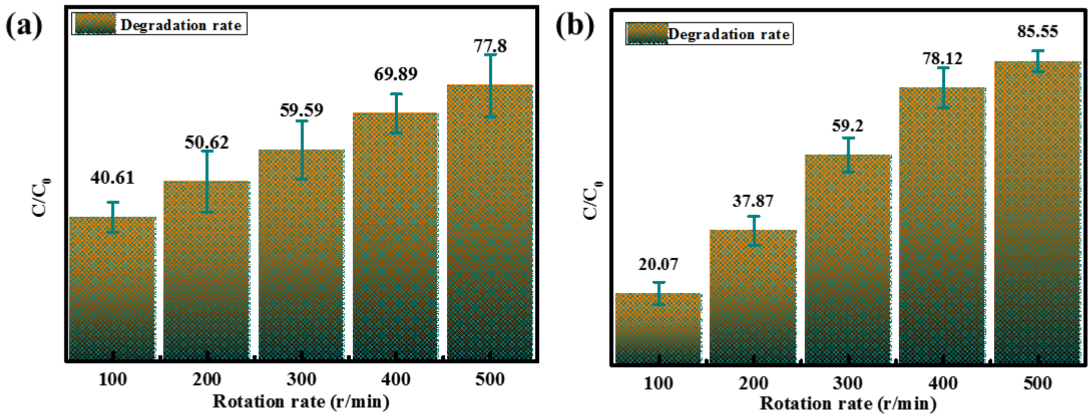

Fig. S4 Influence of different rotation rates on the removal of (**a**) BG and (**b**) DB

Table S5 Degradation intermediates of DB in the presence of TENG and 3DGA@CDs-TNs under visible light irradiation, detected by LCMS in the positive ion mode

	Retention time (min)	Theoretical mass (m/z)	Molecular Formula	Supposed structure
5B	5.259	992.89	$C_{34}H_{24}N_6S_4O_{16}Na_4$	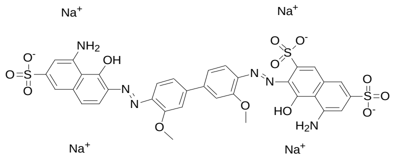
	7.997	903	$C_{34}H_{27}N_6S_4O_{16}$	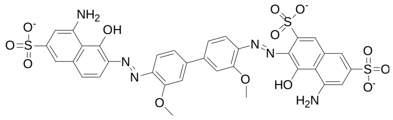
B	6.388	575.58	$C_{24}H_{23}N_4O_9S_2$	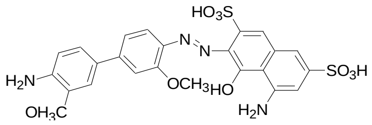
C	6.22	332.29	$C_{10}H_8N_2O_7S_2$	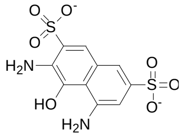
D	5.548	286.27	$C_{10}H_6O_6S_2$	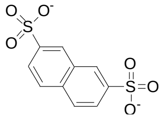
E	1.669	239.25	$C_{10}H_9NO_4S$	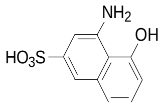
F	4.877	214.26	$C_{14}H_{14}O_2$	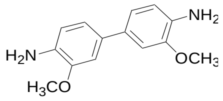
G	4.402	148.11	$C_8H_4O_3$	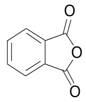
H	3.464	184.24	$C_{12}H_{12}N_2$	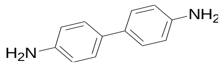
I	1.617	169.22	$C_{12}H_{11}N$	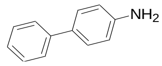
J	5.699	123.11	$C_6H_5NO_2$	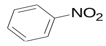

K	6.822	93.14	C_6H_7N	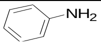
L	6.752	110.1	$C_6H_6O_2$	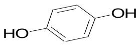
M	0.743	132.11	C_5H_8O	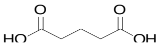
N	11.57	90.03	$C_2H_2O_4$	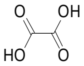
O	0.83	104.06	$C_3H_4O_4$	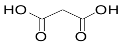

Table S6 Degradation intermediates of DB in the presence of TENG and 3DGA@CDs-TNs under visible light irradiation, detected by LCMS in the positive ion mode

Intermediate products	Retention time (min)	Theoretical mass (m/z)	Molecular Formula	Supposed structure
BR	5.259	482.63	$C_{27}H_{34}N_2O_4S$	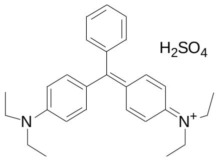
A	6.057	384	$C_{27}H_{32}N_2$	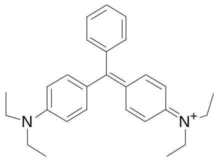
B	3.206	272.12	$C_{19}H_{16}N_2$	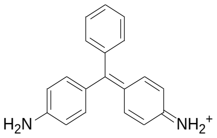
C	1.205	198.21	$C_{13}H_{10}O_2$	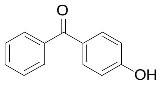
D	1.184	182.22	$C_{13}H_{10}O$	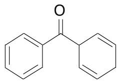
E	1.238	106.12	C_7H_6O	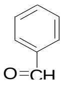

F	1.205	110.1	$C_6H_6O_2$	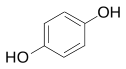<br>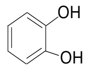<br>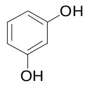
G	1.453	108.09	$C_6H_4O_2$	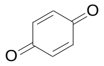<br>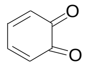<br>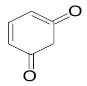
H	1.808	116.07	$C_4H_4O_4$	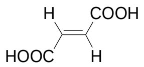<br>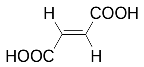
I	0.743	116.17	$C_6H_{12}O_2$	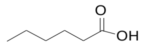
J	2.162	118.1	$C_4H_6O_4$	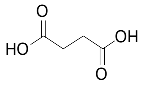
K	1.281	90.03	$C_2H_2O_4$	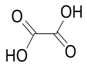
